# Dual-energy computed tomography has limited diagnostic sensitivity for short-term gout

**DOI:** 10.1007/s10067-017-3753-z

**Published:** 2017-08-12

**Authors:** Ertao Jia, Junqing Zhu, Wenhui Huang, Xiaoguang Chen, Juan Li

**Affiliations:** 1 The Department of Internal Medicine of Traditional Chinese Medicine, College of Traditional Chinese Medicine, Southern Medical University, Guangzhou, Guangdong 510515 China; 2 The Department of Rheumatology, Nanfang Hospital, Southern Medical University, Guangzhou, Guangdong 510515 China; 3 The Key Laboratory of Prevention and Control for Emerging Infectious Diseases of Guangdong Higher Institutes, Department of Pathogen Biology, School of Public Health and Tropical Medicine, Southern Medical University, Guangzhou, Guangdong 510515 China

**Keywords:** Clinical trials, Disease duration, Dual-energy computed tomography, Gout

## Abstract

The aim of this study was to discuss the diagnostic value of dual-energy computed tomography (DECT) in patients with gout during different disease phases. Two hundred twenty-one patients (136 with gout and 85 with other arthritic diseases) were recruited to the study. Arthrosis pain was evaluated in all patients by DECT scans. We calculated the sensitivity and specificity of DECT for the diagnosis of gout, including the first onset period, less than 24 months period, and more than 24 months period. We then investigated the related risk factors of urate crystals volume in the foot. The diagnostic sensitivity of DECT in the first onset, less than 24 months, and more than 24 months groups was 35.71, 61.54, and 92.86%, respectively. The overall sensitivity and specificity values were 80.88 and 88.24%, respectively. The multilinear regression analysis showed that longer disease duration (*P* = 0.001) and higher serum uric acid (SUA) (*P* = 0.001) were the two important predictive factors of the monosodium urate (MSU) crystal volume in the foot. DECT provides good diagnostic accuracy for detection of MSU crystal deposits in gout patients. However, DECT has limited diagnostic sensitivity for short-term gout patients, especially for the first onset patients. Longer disease duration and higher SUA were predictive factors of MSU crystal volume.

## Introduction

Gout, which is now one of the most common types of inflammatory arthritis, is caused by monosodium urate (MSU) crystal deposition in joints and tissues. In the USA, 6% of men and 2% of women suffer from gout, and 21% of people suffer from hyperuricemia [1]. The clinical characteristics of gout are hyperuricemia, acute gout flare, tophi, abnormal joints, etc. Besides, several comorbidities are associated with gout, including hypertension, chronic renal disease, and cardiovascular disease [2–5].

Traditionally, the gold standard for gout diagnosis is the identification of MSU crystals in joints or tophi via aspiration. However, aspiration is not regularly performed in daily outpatient clinics [6] and the false negative rate of aspiration has been found to be more than 25% in acute gout patients [7]. Additionally, some small joints are very difficult to aspirate (such as the metatarsophalangeal joint). Recent studies showed that imaging techniques may have a potential role in the identification of MSU crystals, including plain radiography, ultrasonography, MRI, conventional computed tomography (CT), and dual-energy computed tomography (DECT). DECT has been reported to have greater sensitivity (78–89%) and specificity (93–100%) than the other techniques for gout diagnosis [8–11]. Thus, DECT is regarded as a critical appliance to diagnose gout patients [12]. However, most studies presented with a very long disease duration of gout (minimum mean disease duration is 7 years) [13]. To date, there are no reports investigating the diagnostic value of DECT for gout patients with a short disease duration (less than or equal to 2 years). Therefore, the aim of the present study was to evaluate the diagnostic value and clinical significance of DECT for gout with disease duration.

## Methods

### Ethics approval statement

All procedures were approved by the Medical Ethics committee of Nanfang Hospital of Southern Medical University.

### Study patients

Two hundred twenty-one patients who suffered from peripheral joint pain were recruited from May 2013 to December 2016 from the rheumatology outpatient and inpatient clinics of the Southern Hospital in China. Gout was diagnosed based on the 1977 American College of Rheumatology (ACR) criteria as the presence of at least 6 of 12 items (including clinical manifestations, laboratory tests, and X-rays) [13]. Based on the diagnosis of two independent rheumatologists, the patients were divided into the gout group and the control group (with other arthritic conditions). The gout group was not on urate-lowering therapy. Baseline data were collected, including gender, age, disease duration, serum uric acid (SUA), creatinine levels, erythrocyte sedimentation rate (ESR), C-reactive protein (CRP), tophi, and number of gout flares during the last year.

The mean age of the 136 gout patients was 49.15 ± 1.69 years, and 130 (95.59%) of them were male. The mean disease duration was 4.88 ± 0.56 years, the mean SUA was 507.91 ± 16.67 μmol/L, and 110 (80.88%) patients presented with hyperuricemia. The mean number of gout flares during the last year was 4.29 ± 0.35 times. Kidney function was abnormal in 40 patients (glomerular filtration rate ˂90 mL/min). The control group consisted of 85 patients (55 [64.71%] males). The mean SUA was 319.12 ± 19.37 μmol/L, 10 (11.76%) patients presented with hyperuricemia, and 3 patients in the control group had abnormal kidney function.

According to the disease duration, we divided the gout patients into three groups—the first onset, less than 24 months, and more than 24 months groups.

### DECT imaging evaluation

All of the patients received DECT scan of the painful joint. The DECT scans were performed using a dual X-ray tube 128 detector-row CT scanner (Somatom Sensation Flash, Siemens AG, Erlangen, Germany) with simultaneous image acquisition at 140 kV (including an additional tin filter) and 80 kV. All the datasets were reconstructed using both soft tissue and bone kernels with a slice thickness of 0.75 mm and reconstruction increment of 0.5 mm. The bone kernel images were evaluated for erosions.

The tissue characterization of DECT and the detection of MSU crystals were based on the changes in the attenuation of X-rays with variable energies in different tissues and required the post-processing of soft tissue datasets. The MSU crystals were shown in green. Post-processing was performed using a proprietary work station (Siemens Multimodality Workplace, Software version MMWP Syngo CT 2010A, Siemens AG, Erlangen, Germany). The following pre-defined standard parameters were applied for 80- and 140-kV images: the CT value for the soft tissue/fluid was set at 50 HU (Hounsfield units), the threshold ratio parameter for differentiation between calcium and urate was set at 1.36, the minimum parameter controlling algorithm sensitivity was 150 HU, and the maximum parameter for dense bone suppression was 500 HU. Using a dedicated automated volume assessment software, the MSU crystal deposition volumes were measured.

The DECT scans were evaluated by two independent readers with rich experience in automated DECT volume assessments. The readers were also blinded to the rheumatologist’s evaluation. The mean MSU crystal volume was calculated based on the two readers.

### Statistical analysis

Data was analyzed using SPSS (v16; SPSS Inc., Chicago, IL) and GraphPad Prism v5 (San Diego, CA). For continuous variables, the results were presented as the mean ± standard error ($\overline{x}$ ± *s*). Inter-observer reproducibility was assessed by intraclass correlation coefficient (ICC) and limits of agreement (Bland and Altman) analyses. The sensitivity and specificity of DECT for gout with different disease periods were calculated. Linear regression was used to determine significant predictors of MSU crystal volumes in the foot. Factors at 10% significance level were further entered into the multivariate linear regression model, in which a stepwise selection methodology was applied to yield the best model. The serum uric acid and urate crystal volume were compared before and after treatment using a paired *t* test. A two-tailed significance level of 0.05 was used for all tests.

## Results

### The MSU deposition of the painful joint

In the gout group, 110 (80.88%) patients had positive results. Among the lower limbs, the joints were affected in the feet (58.82%), knees (16.18%), and ankles (17.65%). The positive rate was 85.00, 81.81, and 83.33%, respectively. Among the upper limbs, the joints were affected in the hands (7.35%), wrists (4.41%), elbows (1.47%), and shoulders (1.47%). The positive rate was 80.00, 33.33, 100, and 100%, respectively. There were ten patients that had MSU crystal deposition in the foot and ankle. The affected rate was higher in the lower limbs than that in the upper limbs. In the control group, we found that there were ten patients who had MSU crystal deposition in the ankle. There were four patients with hyperuricemia who had MSU crystal deposition, and there were six patients with hyperuricemia who did not (Table 1).

**Table 1:** The MSU deposition of the painful joint

MSU locations		Gout group			Control group		
		MSU positive (*n* = 110)	MSU negative (*n* = 26)	Positive rate (%)	MSU positive (*n* = 10)	MSU negative (*n* = 75)	Positive rate (%)
Lower limb	Foot	68	12	85.00	0	0	0
	Knee	18	4	81.81	0	5	0
	Ankle	20	4	83.33	10	25	28.57
Upper limb	Hand	8	2	80.00	0	20	0
	Wrist	2	4	33.33	0	20	0
	Elbow	2	0	100	0	5	0
	Shoulder	2	0	100	0	0	0

*MSU* monosodium urate

### Sensitivity and specificity of DECT detection of MSU crystal deposition for the diagnosis of gout with different disease durations

The sensitivity and specificity of DECT detection of MSU crystal deposition for gout diagnosis with different disease durations are showed in Table 2. The sensitivity was 35.71, 61.54, and 92.86% in the first onset, less than 24 months, and more than 24 months groups, respectively. The diagnostic accuracy was 75.22, 78.1, and 90.53%, respectively. The total sensitivity, specificity values, and diagnostic accuracy were 80.88, 88.24, and 83.71%, respectively.

**Table 2:** Sensitivity and specificity of DECT for the diagnosis of gout with different disease durations

Group	Positive *n*	Negative *n*	Sensitivity % (95% CI)	Specificity % (95% CI)	Positive predictive value % (95% CI)	Negative predictive value % (95% CI)	Diagnostic accuracy % (95% CI)
First onset	10	18	35.71 (20.71, 54.17)	88.24 (79.68, 93.48)	50.00 (29.93, 70.07)	80.65 (71.47, 87.39)	75.22 (66.52, 82.26)
Less than 24 months	32	20	61.54 (47.96, 73.53)	88.24 (79.68, 93.48)	76.19 (61.47, 86.52)	78.95 (69.71, 85.94)	78.1 (70.46, 84.21)
More than 24 months	78	6	92.86 (85.28, 96.69)	88.24 (79.68, 93.48)	88.64 (80.33, 93.71)	92.59 (84.77, 96.56)	90.53 (85.17, 94.09)
Total	110	26	80.88 (73.46, 86.61)	88.24 (79.68, 93.48)	91.67 (85.34, 95.41)	74.26 (64.95, 81.78)	83.71 (78.27, 88.00)

*CI* confidence interval

### The MSU crystal volume in the foot

**Fig. 1:**
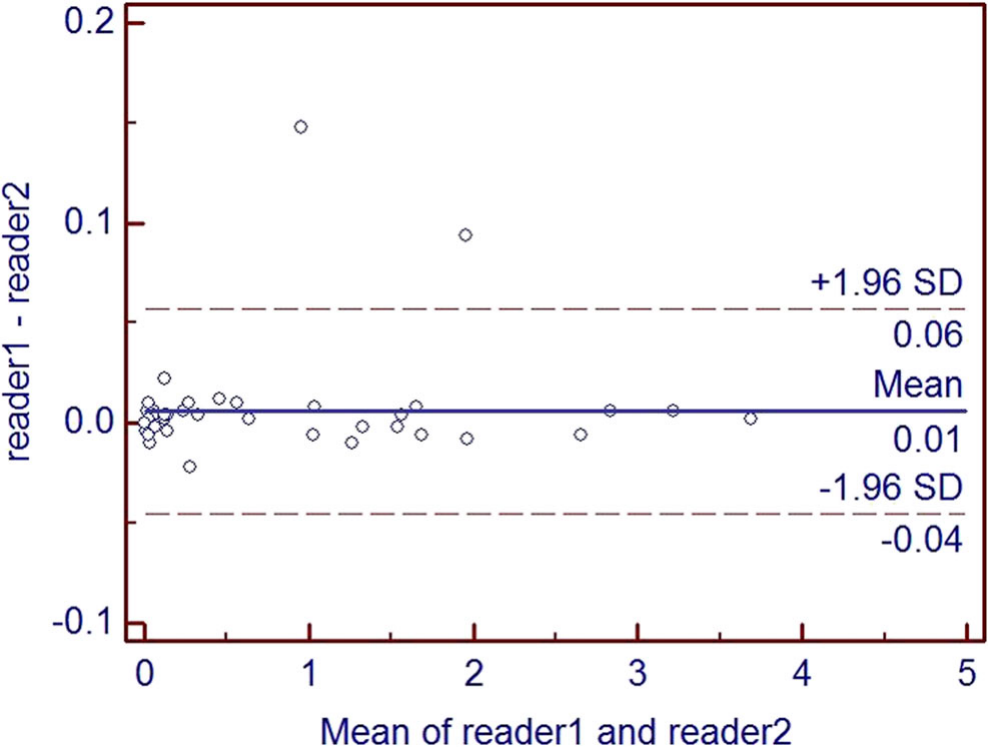
Bland–Altman plots of differences between readers. Differences in MSU crystal volumes measured by two independent readers plotted as a function of the mean MSU crystal volume. The *blue solid horizontal line* represents the mean difference (i.e., bias) in volumes measured by the two readers. The *dashed horizontal lines* represent the observed 95% limits of agreement (−0.04 to 0.06 cm^3^)

The mean MSU crystal volume in 94 patients was 0.685 cm^3^ (range from 0 to 3.689 cm^3^). The MSU crystal volumes were calculated in the foot and ankle. The mean MSU volume that was calculated by the two readers was 0.688 ± 0.099 and 0.682 ± 0.099 cm^3^. The intraclass correlation coefficient between readers was 1.000 (95% CI, 0.999 to 1.000) for all locations. A plot of the differences in MSU crystal volume between readers as a function of the mean volume of the respective MSU crystal deposit showed that the measurement between readers was similar regardless of the MSU crystal volume (Fig. 1).

In the multivariate linear regression analysis, the disease duration (*P* = 0.001), level of SUA (*P* = 0.001), number of gout flares (*P* = 0.031), age (*P* = 0.006), and ESR (*P* = 0.009) were significant predictors of the MSU crystal volume in the foot and ankle (Table 3). In contrast, sex, CRP, tophi, and abnormal kidney function showed no significant results.

**Table 3:** Multivariate analysis for predictors of the MSU crystal volume in the foot

Parameters	*B*	95% CI for *B*	*P* value
Disease duration	0.066	0.028, 0.104	0.001
Serum uric acid	0.002	0.001, 0.003	0.001
Gout flares	0.064	0.006, 0.122	0.031
Age	0.014	0.004, 0.024	0.006
ESR	−0.008	−0.014, −0.002	0.009

*MSU* monosodium urate, *CI* confidence interval, *E* estimate, *OR* odds ratio

## Discussion

The gold standard for gout diagnosis is the identification of MSU crystals in the joints or tophi by aspiration. However, the false negative rate of aspiration is more than 25% in patients with acute gout [7], and it is difficult when applied in some small joints. These limitations will inevitably affect the accuracy of DECT. The classic clinical manifestations of acute gout can be utilized to diagnose gout with a very high sensitivity of 96% (95% CI, 91 to 101%) and specificity of 97% (96 to 98%) [7, 14]. EULAR evidence-based [15] and multinational evidence-based recommendations for the diagnosis and management of gout [16] indicated that the diagnosis of gout can be supported by classic clinical manifestations. The 1977 ACR criteria, which are based mainly on clinical manifestations, were utilized herein. A previous study showed that the sensitivity and specificity of the criteria were 87.6 and 94.9%, respectively [17]. Thus, we utilized these criteria for the diagnosis of gout in our study.

The present study demonstrated that the sensitivity of DECT changes with different disease periods. The total sensitivity was 80.88%, which is lower than that reported in previous studies (93–100%). However, the specificity was 88.24% in this study, which is higher than the previous assessments (78–83%). The present study showed that the sensitivity of DECT was low for the patients with less than 24 months duration, especially in the first onset group. A previous study [11] showed that 43 patients with gout identified by aspiration were recruited, among which 50% of the patients had a disease duration of less than 6 weeks. In that study, four of the false negative patients were in the first onset group. Although the sensitivity was 90% (95% CI 76 to 97%), 28 patients with insufficient synovial fluid samples were excluded. This exclusion would finally affect the sensitivity of the DECT diagnosis of gout. Another explanation for the low sensitivity of DECT for gout with a short duration may be a consequence of MSU crystals that are too small or a MSU concentration that is below the detection limits. Previous studies have reported that DECT can only detect MSU crystal with a minimum diameter of 2 mm [18] and a volume concentration of more than 15–20% [19]. The present findings showed that the disease duration in the MSU-positive group was longer than that in the MSU-negative group.

Serum uric acid and disease duration are the main factors contributing to MSU crystal deposition. Previous studies have suggested that the solubility of uric acid is 6.8–7.0 mg/dL [20], and the quantity of MSU crystals was associated with serum uric acid levels [21, 22]. When the concentration of serum uric acid is consistently lower than its solubility, urate crystals would be dissolved [23]. Huaxiang Wu et al. [24] demonstrated that disease duration was the main contributing factor to MSU crystal deposition, whereas the calculated MSU volume showed no significant result. In the present study, we calculated the MSU volume in the foot, and thus the results could provide better evidence supporting the relationship between disease duration and the volume of MSU crystals.

## Conclusion

DECT provides good diagnostic performance for detection of MSU crystal deposits in gout patients. However, DECT has limited diagnostic sensitivity for gout with a short disease duration, especially in the first onset patients. A longer disease duration and higher SUA were predictive factors of the volume of MSU crystals.
